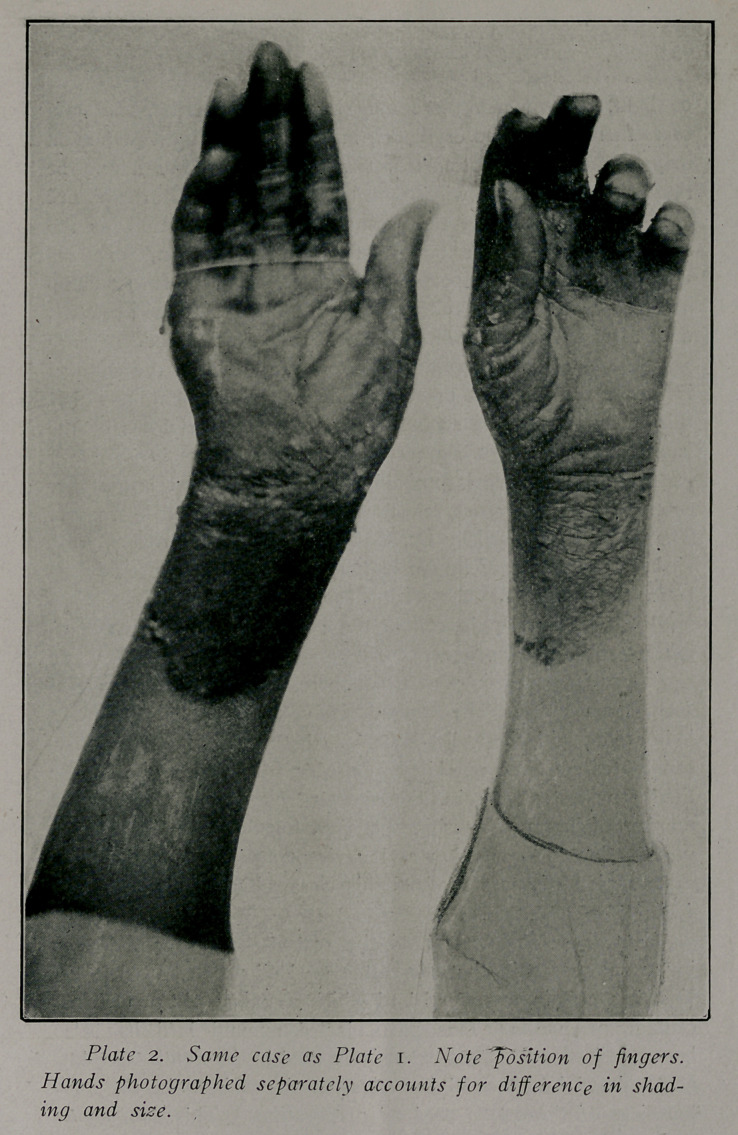# Etiology, Pathology and Treatment of Pellagra

**Published:** 1912-03

**Authors:** Geo. C. Mizell

**Affiliations:** Gastro-Enterologist to Wesley Hospital; Formerly Associate Professor of Physiology and Gastro-Enterology of Atlanta College of Physicians and Surgeons


### Journal-Record of Medicine

Successor to Atlanta Medical and Surgical Journal, Established 1855,
and Southern Medical Record, Established 1870.

OWNED BY THE ATLANTA MEDICAL JOURNAL CO.

Published Monthly

*Official Organ Fulton County Medical Society, State Examining Board, Presbyterian Hospital, Atlanta, Birmingham and Atlantic Railroad Surgeons' Association, Chattahoochee Valley Medical and Surgical Association, Etc.*

EDGAR G. BALLENGER, M. D., Editor.
BERNARD WOLFF, M. D., Supervising Editor.
A. W. STIRLING, M. D., C. M., D. P. H., J. S. HURT, B. Ph., M. D.
GEO. M. NILES, M. D., W. J. LOVE, M. D., (Ala.); Associate Editors.
E. W. ALLEN, Business Manager.

COLLABORATORS

Dr. W. F. WESTMORLAND, General Surgery.
F. W. McRAE, M. D., Abdominal Surgery.
H. F. HARRIS, M. D., Pathology and Bacteriology.
E. B. BLOCK, M. D., Diseases of the Nervous System.
MICHAEL HOKE, M. D., Orthopedic Surgery.
CYRUS W. STRICKLER, M. D., Legal Medicine and Medical Legislation.
E. C. DAVIS, A. B., M. D., Obstetrics.
E. G. JONES, A. B., M. D., Gynecology.
R. T. DORSEY, Jr., B. S. M. D., Medicine.
L. M. GAINES, A. B., M. D., Internal Medicine.
GEO. C. MIZELL, M. D., Diseases of the Stomach and Intestines.
L. B. CLARKE, M. D., Pediatrics.
EDGAR PAULIN, M. D., Opsonic Medicine.
THEODORE TOEPEL, M. D., Mechano Therapy.
R. R. DALY, M. D., Medical Society.
A. W. STIRLING, M. D., etc., Diseases of the Eye, Ear, Nose and Throat.
BERNARD WOLFF, M. D., Diseases of the Skin.
E. G. BALLENGER, M. D., Diseases of the Genito-Urinary Organs.

VOL. LVIII. MARCH 1912 NO. 12

### ETIOLOGY, PATHOLOGY AND TREATMENT OF PELLAGRA.

By GEO. C. MIZELL, M. D.

*Gastro-Enterologist to Wesley Hospital; Formerly Associate Professor of Physiology and Gastro-Enterology of Atlanta College of Physicians and Surgeons.*

### CLIMATOLOGY AND SEASONAL INCIDENCE.

The geographical distribution of Pellagra can not be given with any degree of accuracy. However, the reported areas of incidence show that it has not occurred north of Latitude 51 nor south of Latitude 40. Within this zone it has a wide distribution, and circles the globe both north and south of the Equator.

On the Northern Continent of the Western Hemisphere the disease has been scattered from the northern limit of the United States, from Buffalo, New York, in latitude 42, to Yucatan, Latitude 20. On the Southern Continent it has visited Brazil and Argentine, which extend to Latitude 40.

In the Eastern Hemisphere it has occurred in Poland, Latitude 51, and in every Latitude south until South Africa, in Latitude 30, is included.

The disease, therefore, is not limited to the Tropics, but extends north and south, including a large portion of the Temperate Zone. It will be noted from the above, that the limit in the Eastern Hemisphere, Latitude 51, is much farther north than the limit in the Western Hemisphere, Latitude 42. This geographical difference in position in incidence is fully equalized climatically by reference to an isothermal chart. It will then be seen that the climates of these regions are nearly the same, and are connected by the same isothermal lines. The above latitudes include all regions from which Pellagra has been reported.

The upper borders of the regions of relatively numerous cases are somewhat lower than the lines given above, being latitude 47 in the Eastern Hemisphere, and Latitude 41 in the Western Hemisphere.

While the latitude limit on the Western Hemisphere is about 6 degrees lower than on the Eastern Hemisphere, the same isothermal line connects these situations. Likewise, the southern limit lies on the same isothermal line. We may say then, that endemic and relatively numerous incidence of Pellagra is at present confined within certain climatic limits. This northern climatic limit is the isothermal line—70 degrees F. for July. So far as has been reported, there are no regions of relatively numerous cases south of the Equator, but some cases have been reported from the regions of the isothermal line of 70 degrees F. for January, which is the corresponding month climatically for July in the Northern latitude. Situated between these isothermal lines is about one-half of the inhabited land of the globe—therefore, the disease has a wide distribution, and it has occurred in every latitude within these lines. It is to be understood that the temperature does not have any direct bearing on the development of the symptoms, but that the intensity of the sunlight that prevails in the zone between the isothermal lines is thought to be the main influencing agent. Thus it is not material how high the temperature so long as there is no exposure to the light which produces the temperature. While this is true in regard to the development and progress of the skin symptoms, it is probable that the temperature of the surrounding atmosphere, independent of light, greatly influences the progress of Pellagrous individuals. Whether these patients are exposed to the light or not during warm weather their constitutional symptoms are greatly aggravated.

It is probable that the disease has not occurred to any extent except under the influence of a temperature of 70 degrees F. or higher. Certain it is that it has not been reported to any extent, except within this influence, and yet there is positive evidence that it can develop even when the temperature is around 30 degrees to 40 degrees F., and then during a cloudy period of weather when there is no possible exposure to the sun's rays.

Plates 1 and 2 reproduce pictures taken on February 7th, 1912. A history of the case demonstrates that under some conditions the disease may manifest the typical lesions uninfluenced by season. This is a case of Dr. J. C. White, with whom the writer was associated as Consultant.

Mrs. G., aged 42. Residence, Atlanta 7 years. Weight 160 pounds. Height 5 feet, 8 inches. Previous good health. No prodromal symptoms. On December 16th began feeling sick and complained of general weakness, loss of appetite and dizziness. There appeared upon the back of the hands a dermatitis which was confined to the areas that show normal in Plate 1.

There was a free flow of saliva of salty, tenaceoues nature. After a few days' treatment by Dr. J. C. White these symptoms abated and the patient continued in fairly good health until January 11th, at which time the dermatitis reappeared, but the location was changed. The regions now involved were the backs of the fingers, thumbs and wrists. The condition during the first few days resembled an erysipelatous inflammation. The salivation and stomatitis were now marked. The mucous membrane of the mouth presented the appearance of a general inflammation. The buccal mucous membrane, the buccal surfaces of the gums and the under surfaces of the tongue, were covered with a white membrane. Nausea and vomiting were constant symptoms. The bowels were in fair condition, only three or four soft stools daily. Temperature, 101 degrees, pulse, 120, and fairly good quality. Sleep was much broken by the constant necessity of spitting.

Patient was put upon treatment and diet and confined to a dark room. The dermatitis gradually spread until the areas shown in the plates were involved. The nausea and vomiting were relieved at once. In about one week the patient was in imminent danger from weakness of the heart. Heart sounds were foetal in character. Pulse 160, and very weak. Prostration was marked. There was a marked wrist drop, especially of the left hand, and there was inability to use the hands or fingers except with difficulty.

During this time there had developed a dermatitis on the feet, knees and elbows, and a vaginitis with oedema and irritation of the skin and mucous membrane covering the vulva and surrounding regions. This type of case is usually rapidly fatal. These symptoms gradually subsided and the patient in a few days was out of danger. On February 7th the patient was sufficiently improved to allow photographing the hands. The dermatitis was of the "moist" variety and as the inflammation began to subside large blebs were formed. Desquamation, which took place in large flakes is well shown in the plates.

The interesting points in this case are: 1st. Season of incidence; 2nd. The acute nature of the attack; 3rd. The good physical condition of the patient; 4th. Two attacks of dermatitis in one month; 5th. Well marked new areas of dermatitis in second attack. In these features the case does not accord with the accepted ideas of the disease and neither does her prompt recovery.

This is one of several typical cases occurring during the winter that has come under my observation.

However, it is a fact generally accepted, that practically all of the cases of Pellagra develop only when the temperature is near or higher than 70 degrees F., and it is generally accepted that the symptoms are influenced by sunlight. With all due respect to the adherents of the Toxico-chemic theory, it is difficult to understand how the action of a poison introduced from without is going to be held in abeyance until a certain seasonal incidence brings out its action.

It is impossible to harmonize the seasonal incidence of Pellagra with the action of any known poison. Grant that "Pellagrosine," extracted from spoiled corn, is the specific poisonous substance of spoiled corn, and together with the red oil of spoiled corn is analogous to Ergot, and you will be driven to the conclusion that the symptoms produced should be analogous to ergotism in that poisons of such character are independent of climatic influence. There is no seasonal incidence in Ergotism, Alcoholism, or any other Toxico-chemic poison. Beri-beri, to which Pellagra has been compared, has no seasonal incidence. Here the writer wishes to emphasize the possibility of spoiled corn being a cause of Pellagra. At the same time, no case of Pellagra has ever come under obserativon that a satisfactory investigation did not show the consumption of Linolin in some other form.

---

What seems to be a satisfactory explanation of such unusual cases as the above is, that these cases are in reality toxico-chemic in their origin as far as secondary cause as concerned. This patient had been eating a certain cooking oil which may have been pressed from unsound seed, and the corn product that she was eating at the time of the attack was known to be unsound. Thus she may have consumed the toxico-chemical poison which, upon its introduction into the body, set up the dermatitis and salivation. As we shall see later, the poisonous agents in spoiled corn are probably oxidation products of the oil of corn.

It will be recalled that it has been shown that linolin and linolic acid compounds are deposited in the various tissue as such, and that they quickly undergo oxidation; also that when old or exposed oil is added to the fresh oil, the rapidity of oxidation is greatly increased. Under these conditions, it is possible that we have a bichemical poison developed as the result of the introduction into the body of a toxico-chemical poison. This would simply be a substitution of the toxico-chemic poison as a secondary exciting cause for sunlight, which is the usual and common secondary cause.

---

Dr. Sambon sets forth certain conditions in support of his fly theory, which, if true in Italy, are certainly not true in the United States. One of the these conditions is, that Pellagra shows a definite season incidence, spring and fall.

Opinions on the different clinical features of Pellagra are hopelessly divided. As in every other proposition, when the correct theory is recognized and applied, every phase will be easily explained.

Accept the theory that the oxidation products of Linolin are the cause of Pellagra, whether these oxidation products are from the linolin of corn, cottonseed oil, oil of walnut or sunflower seed; and that these poisons may produce the disease, whether the poison is *preformed* outside of the body, or whether it is developed as a biochemical poison. Then it will be possible to harmonize the apparently contradictory facts in the history of Pellagra. Many important statements made by various writers do not accord with the opinion of the writer, or of other writers. In presenting these opinions and observations of others, the writer does not presume to express an adverse opinion, except as borne out by clinical experience. To those who have read the preceding articles it will appear that he had accepted the seasonal incidence of Pellagra with the sun as the only exciting cause of the dermatitis. Clinical experience has since altered both of these views. At first, the new clinical experience appeared to be fatal to this theory, but mature consideration has brought to light conclusions which have been a great aid in understanding several phenomena.

Strict adherence to the bio-chemical idea of the disease is compatible with seasonal incidence, and the chemical (oxidizing) influence of sunlight as the only secondary cause of the dermatitis, but incompatible with the development of certain lesions in midwinter. When it is admitted that the effect of a poison is the same whether it arises within or without the body, the explanation of winter incidence of the disease is clear.

As opposed to a strict season incidence, limited to spring and fall months, development of the dermatitis in 100 cases is given below:

| | | | |
|---|---|---|---|
| January | 1 | July | 26 |
| February | 1 | August | 11 |
| March | 2 | September | 6 |
| Aril | 14 | October | 1 |
| May | 17 | November | 0 |
| June | 19 | December | 2 |

Dr. Sandwith, in his Diseases in Egypt, states that in 200 out of 300 cases the dermatitis occurred in January and February. Egypt is in the same latitude as Florida, but the climate is somewhat different.

The table shows that the number of cases in developing the dermatitis gradually rises in number until August 1st, and then rapidly declines. There is no spring and fall incidence here, which is so much insisted upon by some. The same will be noted in Dr. Sandwith's cases.

If there is a spring and fall incidene in any region it may be explained by climatic influences or relative exposure. It should be borne in mind that the dermatitis does not appear immediately after one or two days' exposure. In most cases it requires several days, or several weeks, according to the intensity

*Plate* I, *Atlanta Case. Dermatitis began Jan.* 11, 1912

*Photographed Feb.* 7, 1912.

*Plate 2. Same case as Plate 1. Note position of fingers. Hands photographed separately accounts for difference in shading and size.*

of light, after which there may be a latent period. In mild cases there may be no erythema, in which case the desquamation may be the first symptom to attract attention. Thus it may be that considerable time will elapse between the period of exposure and the appearance of the dermatitis or desquamation, as the case may be. This period of latency or "incubation," explains why many cases are reported as having occurred during a damp or rainy season, also why many cases do not show the dermatitis until after they have been confined to the house or bed.

Abundant clinical evidence favors the idea that the climatic influence is dependent upon the intensity of sunlight, and that this intensity must be such as prevails within the limits of the isothermal lines of 70 degrees F. for midsummer.

While the above is probably true of by far the largest per cent. of cases, there are few that occur uninfluenced by season. The question is asked by Dr. C. C. Bass, of New Orleans, why, if the consumption of linolin or its products is the cause of Pellagra, the disease has not occurred in certain eastern cities, New York City, Philadelphia, etc., as these regions have been consuming cotton seed oil products.

In reply, it is pointed out that there are a few cases reported from these cities. The comparatively small number of cases is in accord with the well known fact that the people of the Eastern States consume notably less greasy food than those of the Southern States. Again, the shorter summer and the fewer number of hours of sunshine per day, together with limited exposure, due to occupation and environment, would permit the consumption of more food of this class without deleterious effect.

But these are not all of the reasons. Perhaps the most important reason is, that the cotton seed oil product used in the East is a certain compound lard which contains only a small per centage of linolin. This particular compound lard has also been, until recently, most extensively used in the South, and data accumulated by personal investigation shows it to be the most harmless of the cotton seed oil preparations. This data shows that preparations of this class may be consumed in relatively large amounts for a long period of time before giving symptoms.

In recent years, in the South there has come into general use a cotton seed oil which contains a large per cent. of linolin; and it has been extensively used for making dressings, as a salad oil, as a cooking oil, and quite a large number of people have been "seasoning" vegetables with it. Another fact that enters into this regional incidence is, that the South is a large consumer of corn, which adds somewhat to the total quantity of oil ingested.

Dr. Bass asks the same question in regard to Germany, France, Turkey, Denmark and Sweden.

As Turkey and France are the only countries he has named that come within the climatic zone, they can not be eliminated on climatic grounds. Only Southern France comes within the climatic zone, and until very recently the disease was quite prevalent in this region, and is still prevalent to an extent. That he may be in error as to the increased consumption of this class of oils in France is quite probable, as shown by the decrease in importation of these oils into France. Also, there has been a deided increase in the consumption of "Vegetaline," made from cocoa bean, shipped from Central and South America. The amount of cotton seed oil imported into France amounts to only a little more than half the number of gallons of olive oil and peanut oil used in the Nantes district for packing sardines. A cheap grade of olive oil made from cotton seed oil is used for cooking purposes in southern France, from whence Pellagra is still reported to some extent. From the above it will be observed that the amount of cotton seed oil consumed is a very small per cent. of the fats consumed in France. Another reason for the decrease of Pellagra here is that corn is not consumed to such a great extent as formerly, thereby further diminishing the amount of these oils ingested.

In Turkey, the importation of cotton seed oil for edible purposes was prohibited until May, 1907. Before this date other oils were used to some extent and Pellagra was known in Turkey some years before cotton seed oil was imported. Turkey is the largest olive oil producing country in the world. Statistics brought up to date would probably show a parallel incidence of the disease and the consumption of linolin containing oil.

The greater part of Germany, and all the other countries named, are far above the climatic zone. It is true that some cotton seed oil is imported into these countries, but how much and for how long? What part is used for industrial purposes, and what part for edible purposes? How much oil, and in what proportion to other fats is it consumed per individual? Germany is not an oil consuming nation, and the amount of linolin containing oils consumed is insignificant as compared with the non-drying oils. The cheapest cooking grease in Germany is composed of cocoanut oil, for which there is a big demand.

In Sweden, of the 74,370 barrels of comestible oils imported in 1908, 14,300 were cotton seed oil. This was used in the manufacture of margarine. In the proportion used and in the climate of Sweden this product would probably be harmless.

The climate of Denmark is practically the same as Sweden. Here the raising of hogs is an important industry. Pellagra is unknown in countres that adhere largely to animal fat for food purposes.

Holland and Norway consume some cotton seed, but there is nothing to show that these people are oil consumers, and if they are, the climatic influences would be against the development of the characteristic symptoms. Hence, the disease might occur without recognition.

The above data in regard to food products above mentioned is obtained from the Daily Consular and Trade Reports.

Dr. Bass has eviently expressed an opinion on the subject without due consideration of its basis. To say that the disease from the standpoint of history may as well be attributed to canned goods, soft drinks, etc., is unworthy of his able mind, and when he is fully informed of the theory and its basis he may decide that the few words which he thought would set it aside are utterly inadequate. His attack upon the admissibility of the results when animals are fed upon cotton seed products will be fully discussed in due order.

PERIOD OF INCUBATION.

As to a period of incubation which is so necessary to any theory based on a living virus or organism, it appears that clinical evidence is entirely opposed. Furthermore, the dermatitis of Péllagra, disappearing as it does to recur again in one to twelve months, absent in some cases, the initial lesion in some cases, or a late incident in others, and appearing at any period of the disease, is not in accord with infectious, eruptive disease. Dr. Sambon gives instances where cases residing in the supposed endemic areas, after contracting the disease, moved to "non-Pellagrous" regions. Notwithstanding this change of residence, the attacks recurred each year. In these cases there must have been, according to his ideas, a period of incubation each year, but he offers no suggestion as to how reinfection took place. This recurrence and annual "period of incubation" without reinfection is not in accord with the course of infectious eruptive diseases.

Nor does the eruption in eruptive diseases present such a varied pathological picture. Here the dermatitis presents the most extreme difference. Thus it may resemble an erysipelatous inflammation or only a slight sunburn. It may persist for many months, or it may disappear in a few days. There may or may not be pigmentation. Desquamation may cover a period of many months, or may be complete in a few days. By its effects the skin is thickened; again it may be thinned. In most cases there is a seasonal incidence dependent upon sunlight, but rarely; the dermatitis may appear independent of light exposure.

While we have come to look upon the dermatitis as pathognominic of the diesase there are several skin diseases that appear in susceptible individuals under the influence of light energy. In Eczema Solare. the dermatitis appears in the spring and is limited to the exposed surface. This, and other skin diseases, begin as a Solar-Erythema, and can not be differentiated from the dermatitis of Pellagra.

According to Bouchard, Pellagrous dermatitis is only a Solar-Erythema developed in a Pellagrous subject. By experiment he has shown that it is due to Solar light. It does not form an integral part of the Pellagrous syndrome but is produced by virtue of a general state, involving, as it must, a malnutrition (perverted nutrition would be more applicable) of the skin as well as rendering it susceptble to the untoward influence of light.—Light Energy, Cleaves.

We can not doubt Bouhard's experiments and his conclusions, but he probably did not see such cases as Mrs. G. How, then, may we explain these cases? If it is admitted that oxidized oil of corn (or any other oil of this class) is poisonous, whether it is oxidized inside of the body or out of it, the explanation is simple.

In admitting the above it must be borne in mind that it is possible that the oxidized oil or the unoxidized oil may be perfectly harmless when consumed within certain limits. And moreover, like many other poisons, it may even be beneficial within certain physiological limits and become deadly by overdose.

After the experience of the inhabitants of the Southern States no one would argue that sound corn is unwholesome when varied with other food, or that the oil it contains would be deleterious in the proportion that it exists in the grain, but add to this a corn oil or a cooking oil that contains double the percentage of the fat common to this class of oils and exclude other fats. Now, in addition to this one class of fats eat "Western" (spoiled) corn for one week or one month. The elastic human organism may escape all of these conditions, but will it always do so?

To show that there has been no unwarranted assumption in stating that oxidized oil of corn is poisonous, the following is quoted in full from "'Pellagra," Marie, translated by Lavinder and Babcock.

"The rancid oil of corn has produced various results, physiological and toxic. These results have been confirmed by the investigation of Biffi. They are diminution in weight, and increase in temperature. In frogs, temporary torpor was produced, with injections of two grams. The effects are variable, according to the quality of the corn used in making the oil. Control experiments have been made by injection of olive oil, and oil from fresh and sound corn. The results were negative."

For the present this evidence that oxidized oil of corn is poisonous will suffice. More will be forthcoming later.

Cleaves, in discussing the dermatitis in Pellagra, says: "In this condition it is not a sensative skin abnormally influenced by light, but a diseased skin upon which the light acts to complicate the pathological condition. It is first an erythematous cutaneous alteration, then desquamating, and is associated with a marked cachexia. There is great emaciation, digestive disorders or pshychic manifestations. The erythema of pellagric patients is considered the most characteristic and constant symptom. It follows the uncovered portions of the body and only comes on when the subject is cachectic, never as an initial phenomena. In the beginning it seems like a solar erythema, coming on in the spring and disappearing in the autumn."

It has been shown that the adipose tissue and the cellular fat and fat-containing compounds entering into the tissues of the body may partake of the nature of the ingested fat and thereby become unstable in composition.

According to the same facts already set forth it may be presumed that the fat of the skin and mucous membrane, together with the fat and free fatty acids of sebum and sweat may also be altered in the same manner. If this is true, then the explanation of the effects of sunlight on the skin in these cases need no longer remain a mystery.

According to McLeod, in his Pathology of the Skin, the existence within the horn cells of fat, which Ranvier has compared to beeswax, makes the stratum corneum a water proof coating to the body, acting as it were, as an impermeable varnish over the skin and preventing the absorption of water and substances dissolved or suspended in it.

Accoring to the same author, the cells of the stratum lucidum stain with osmic acid, showing that they contain fat. In addition to this fat of the skin the sebum and sweat contain fat and fatty acids. The great importance of fat in mantaining the integrity of the epidermis is well known and an unstable composition of fat would bring about just such a condition of the skin as would explain the phenomena of these lesions.

Relating to the pathology of Miliaria, S. Pollitzer (Morrow's Dermatology) states: "I have sought (loc. cit.) this factor (individual immunity) in the different degrees, in different individuals, and in different parts of the body, to which the horny layer is impregnated with fat; cells permeated with fat will not absorb water, will not swell and will therefore not occlude the ducts. And we know that in regard to its natural lubrication, the skin in the different parts of the body and in different individuals varies greatly; it is well known that in the negroes and in Southern people generally the skin is particnularly well oiled. It seems likely that the cells of the horny layer (in certain individuals), bathed in erspiration, fail to conify completely, imbibe water and swell up, close the orifice of the ducts."

There is some evidence that keratin and the outer portion of the horn cells are of a fatty nature to which the outer layers of the stratum corneum owes its properties of resistance to water, acids, and resistance to peptic digestion, while being disintegrated by weak alkaline solutions. It is not uncommon for Pellagrins to attribute the dermatitis to various local applications, such as washing powders containing alkaline substances and liniments containing ammonia. There may be more truth in this statement than has been accorded them.

One of the most common and one of the first symptoms present in Pellagra is anidrosis, which may be general or limited to the exposed surfaces. There is also a marked change in the sebum which permits of rapid drying, as shown by the formation in some cases of comedones, which block the sebaceous glands, especially about the face. Not infrequently these comedones in pellagrins are the cause of inflammation, which is characterized as pellagrous dermatitis.

When it is recognized that the ingested fat does influence the cellular structures of the body, and there is much to suggest that it may, we may find a simple explanation of all of the symptoms of this disease. Thus the skin through changes induced by the ingestion of an unstable fat may itself become unstable and be susceptible to influences that the normal skin resists.

To explain the seasonal incidence of the skin symptoms it is only necessary for the skin to become unstable through perverted fat nutrition. Such a skin would also be affected by alkaline applications and the irritating influences of perverted fat excretion of sebum and sweat as it passes between the cells of the altered epidermis. The dryness of the skin of these patients show that the secretion of sebum and sweat is either depressed or stopped in the epidermis. Probably there is both depression and accumulation with evaporation of the fluid of these excretions, leaving behind the solid constituents which contribute to the pathological anatomy of Pellagrous dermatitis.

As far as the skin lesions are concerned, an unstable nature of the fat and fatty compounds would render the skin unstable, and with the advent of summer or hot days with the necessity for increased perspiraion and sebaceous excretion it would be interesting to know what influence the suspension of these functions would have upon the skin and general health.

Such a conception is much more in harmony with the clinical manifestations than a toxico-chemical or parasitic view of the disease.

### Urban Incidence.

To any investigator or physician who is informed on this point, no discussion is necessary.

Another of the conditions necessary to the parasitic theory of Sambon and one cited in its support, is that the disease is limited to certain rural places, and especially to the vicinity of certain streams of water which are inhabited by the Simulium Reptans, and that the disease is limited to certain classes of laborers, the field laborers. As the Simulium does not enter towns or villages, he holds that the disease is not contracted outside of rural districts.

Of 174 cases among my records, 118 are urban and 56 rural. Of the 118 cases, 58 are residents of the city of Atlanta. Several cases who had recently moved to the city are not classified as urban cases. Many of these cases had not been out of the city for years. It is worthy of note that many cases have developed in institutions, viz., asylums for the insane and orphans' homes. Among my cases are some from almost every walk in life, and it does not seem that this is peculiar to my cases.

Dr. K. H. Beall (J. A. M. A., Nov. 18, 1911), gives some very interesting data, relating to the conditons in Texas, from which he concludes that the etiological agent is some thing or condition which exists in the home or around the house and may be active in the day time only. This, it will be noted, is directly opposed to the Italian version of Sambon, and is based upon the fact that his investigations show that the women who remain at home in the day time constitute a large per cent. of the cases.

He also says that "Texas has country which it does not take a vivid imagination to compare with the green valleys of Georgia or Italy; but there are regions where a running stream does not exist for miles, and in these places pellagra is prevalent. In spite of a drougth for the last three years, during which there were few running streams in Texas except a few large rivers, pellagra has markedly increased." He has procured evidence on the position of residence relative to streams in fifty-four cases, and found that but four patients lived near a stream or less than one-fourth of a mile, nine lived one-fourth to one-half mile, while forty-one lived at least one mile; two lived eight miles, four ten miles, one twelve miles, one fifty, and one sixty miles from any overground collection of water; general average of over four miles.

Some months past Dr. G. A. Zeller, of Peoria, Ill., prompted by economic alarm, advised against hasty conclusions. This article contained the inference that cotton seed products could be exclued as an etiological factor in Pellagra by a report which was soon to be made by a committee appointed to investigate the conditions at the Peoria Insane Asylum. This report, which was said to be ready for publication at the time his article was written, has not, as far as can be ascertained, appeared in print.

It is very evident that the best defence Dr. Zeller could have presented for cotton seed oil was to have stated that it had not been consumed by the Pellagrins of that institution. It is to be suspected that the fat used there is some form of cotton seed oil product or he would have so stated.

401 Empire-Life Bldg.

## Figures and Tables

**Plate 1. f1:**
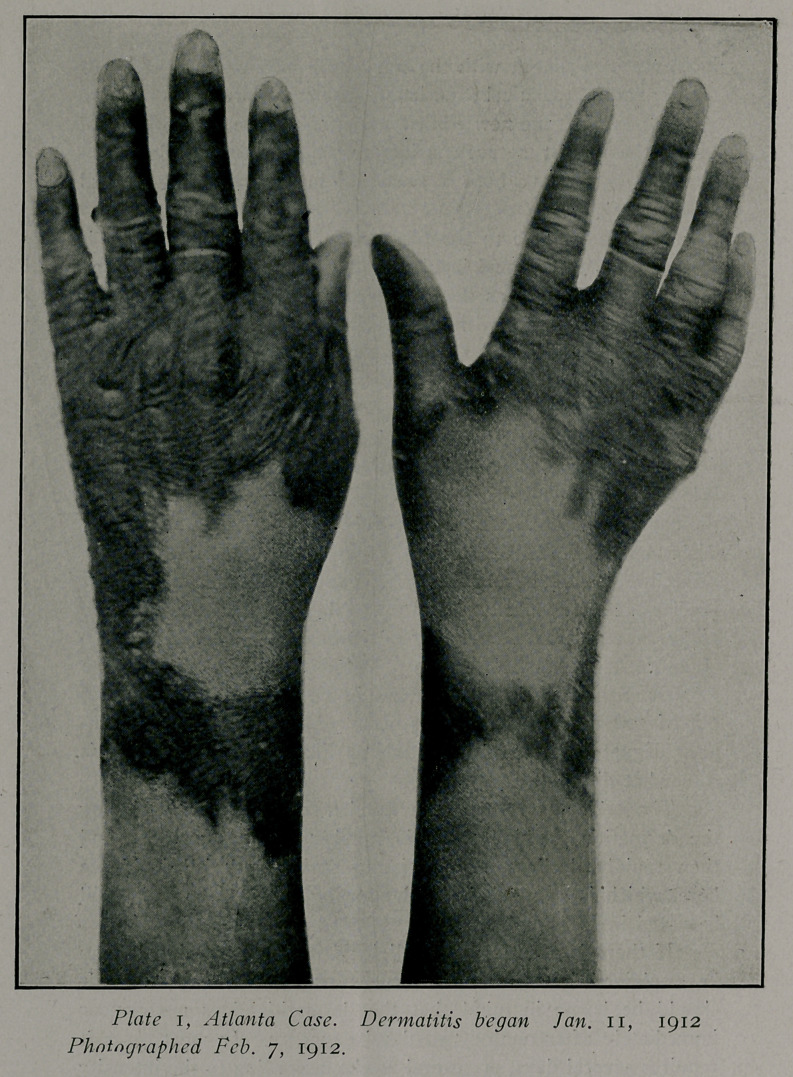


**Plate 2. f2:**